# Electrochemical Redox Cycling with Pyrolytic Carbon Stacked-Layer Nanogap Electrodes

**DOI:** 10.1021/acsami.4c18998

**Published:** 2025-02-19

**Authors:** Nicolai Støvring, Arto R. Heiskanen, Jenny Emnéus, Stephan Sylvest Keller

**Affiliations:** † National Centre for Nano Fabrication and Characterization, DTU Nanolab, Technical University of Denmark, Kgs. Lyngby 2800, Denmark; ‡ Department of Biotechnology and Biomedicine, DTU Bioengineering, Technical University of Denmark, Kgs. Lyngby 2800, Denmark

**Keywords:** redox cycling, reactive ion etching, atomic layer deposition, pyrolytic carbon electrode, generator/collector, differential cyclic voltammetry, multistep chronoamperometry

## Abstract

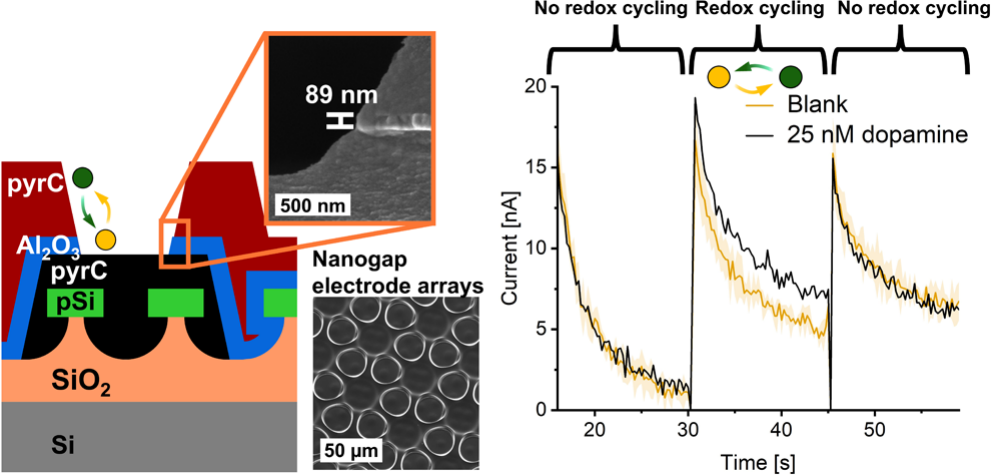

Redox cycling (RC) amplification has been introduced as an efficient strategy to enhance signals in electrochemical sensing at low analyte concentrations of relevant biomarkers such as dopamine. RC amplification requires closely spaced and electrically separate electrodes, preferably with nanogaps. The aim of this study was to establish a method enabling the microfabrication of carbon-based stacked-layer nanogap electrodes (SLNE) designed for RC amplification. Pyrolytic carbon was employed as the electrode material and Al_2_O_3_ deposited by atomic layer deposition as the insulating layer in between the two electrodes. SLNE with 89 nm nanogaps were realized without the need for high-resolution lithography methods, and access to the bottom generator electrode was enabled by dry etching of the insulating layer. Electrical separation between collector and generator electrodes was confirmed using resistance measurements, cyclic voltammetry, and electrochemical impedance spectroscopy. Different SLNE designs and redox cycling modes were investigated in terms of capacitive background current, amplification factors, and collection efficiency using the neurotransmitter dopamine as model analyte. A redox cycling mode, here termed differential chronoamperometry (DCA) combining chronoamperometry with differential cyclic voltammetry, was proposed to minimize the effect of background current drift while still operating with steady-state currents. With DCA, a limit of detection (LOD) of 21 nM, a sensitivity of 83 nA μM^–1^, a linear range from 25 nM to 10 μM, and actual detection at low concentrations of 25 nM were demonstrated for dopamine.

## 1 Introduction

In electrochemical sensing, the oxidation or reduction current of a redox active molecule is recorded at an electrically biased electrode. Electrochemical detection of biomarkers remains challenging due to low concentrations and presence of interfering compounds.^1−3^ A typical approach to increase the current signal is to increase the surface area of the working electrode.^4,5^ A different strategy is redox cycling (RC) amplification,^6−8^ which enhances the sensitivity and selectivity for detection of electroactive molecules such as dopamine.^9,10^ In RC, the current generation from a single molecule can be significantly amplified and large amplification factors have been demonstrated.^11,12^ The amplification occurs due to repeated oxidation and reduction of the molecule at closely spaced, electrically separated electrodes. The electrodes in an RC system are commonly referred to as the generator electrode (GenE) and the collector electrode (ColE). If a molecule is initially present in the solution in reduced form, the GenE “generates” the oxidized form, which then is reduced back to its initial state if it is “collected” by the ColE. Typically, the ColE potential is fixed during measurements, while the GenE potential is stepped or swept.^12^ RC amplification has been used to study electrical double-layer effects,^13^ for single-molecule detection,^14^ and to increase the sensitivity and selectivity of sensors and biosensors.^15,16^ Furthermore, RC is inherently selective against nonreversible redox active species.^10,17^ Dopamine is often employed as model analyte in RC since it is a clinically relevant biomarker, an electroactive molecule, a surface-sensitive reversible redox probe, and its detection is challenging because oxidation products are very reactive resulting in electrode fouling.^18−21^

The time required for a redox molecule to travel back and forth by diffusion between the two separately biased electrodes significantly decreases as the gap decreases with RC current scaling correspondingly as the distance becomes smaller.^6,7,22^ If the gap is reduced to the nanometer regime, the molecule can be recycled several thousands of times in a second. Therefore, metal nanogap electrodes have been fabricated for RC amplification.^23,24^ However, the fabrication of lateral nanogaps is challenging due to diffraction limits in lithographic pattern definition, and methods such as e-beam lithography are typically required.^25^ Another approach is to define the nanogap vertically, which is done for nanocavity systems^26^ or porous stacked-layer nanogap electrodes (SLNE).^27^ In the stacked-layer configuration, the nanogap distance between a bottom and top electrode is defined by the thickness of an insulating spacer layer in between. After deposition of the layer stack, holes are typically patterned into the top electrode, serving as the ColE and the insulating layer by etching processes. This provides analyte access to the bottom electrode functioning as the GenE. It has been shown in several studies that increasing the number of pores increases RC amplification.^11,27,28^ In the recent literature, authors have demonstrated LOD ≤ 100 nM with RC amplification strategies, showing its excellent performance in the detection of low analyte concentrations.^29−31^

So far, SLNE has typically been fabricated using planar thin-film metal electrodes. Photoresist-derived pyrolytic carbon (PyrC) offers high mechanical strength,^32^ chemical inertness,^33^ a wide electrochemical potential window,^2,34^ and highly tailorable electrode geometries.^35,36^ Compared to metal-based SLNE, PyrC SLNE offers an alternative material platform that offers increased flexibility in electrode geometry and processing capabilities such as the inclusion of specialized adhesion structures, in addition to potentially being more suitable for the detection of specific biomolecules such as dopamine.^2^ However, so far PyrC electrodes with nanogaps have only been achieved using single nanofibers,^37^ which is of limited relevance for RC amplification due to the very low total electrode surface area. To date, there have been no reports of PyrC-based SLNE.

In metal-based SLNE, typically SiN_*x*_ or SiO_2_ have been deposited as insulating spacer layers.^38^ Al_2_O_3_ deposited by atomic layer deposition (ALD) is an interesting alternative to these materials, offering conformal coverage of the underlying substrate with a precise control of thickness at the atomic scale,^39^ good insulating properties with a high voltage breakdown strength, and inhibition of carbon diffusion, which negatively affects insulating properties.^40,41^

Here, we for the first time demonstrate the design and fabrication of PyrC SLNE with an open geometry and large arrays of nanogaps in parallel and demonstrate its RC amplification capability for the detection of dopamine at low nM concentrations. The 89 nm nanogaps achieved are, to the best of our knowledge, the smallest gaps demonstrated in a PyrC-based platform for electrochemical sensing.^42^ The nanogaps between two stacked layers of PyrC electrodes were achieved using a layer of Al_2_O_3_ deposited by ALD that allows precise control of the nanogap thickness. UV photolithography and reactive ion etching (RIE) were employed to define microholes in the insulating film providing analyte access to the bottom electrode. Moreover, nanogaps were achieved on a nonplanar topography potentially resulting in larger RC amplification.

In the past, several electrochemical techniques such as cyclic voltammetry (CV) and chronoamperometry (CA) have been employed for the characterization of the performance of RC amplification platforms. Differential cyclic voltammetry (DCV) has been proposed as a promising technique to augment the abilities of RC systems to selectively detect a mix of reversible redox probes.^43,44^ However, only few studies have compared DCV and standard RC techniques.^45^ In our study, we evaluate the performance of the developed PyrC SLNE platform using different RC modes and compare the modes in terms of RC capability and background noise level using the model analyte dopamine (DA) in an open system. Finally, detection of DA at low concentrations down to 25 nM was demonstrated using a novel differential RC mode defined as differential chronoamperometry (DCA) combining CA with DCV. This is among the lowest concentrations detected in recently reported studies on RC amplification-based detection,^31,46^ as well as for novel non-RC systems such as fast-scan cyclic voltammetry.^47^

## 2 Results

### 2.1 Microfabrication of Pyrolytic Carbon Stacked-Layer Nanogap Electrodes

#### 2.1.1 PyrC SLNE Design

**Figure 1:**
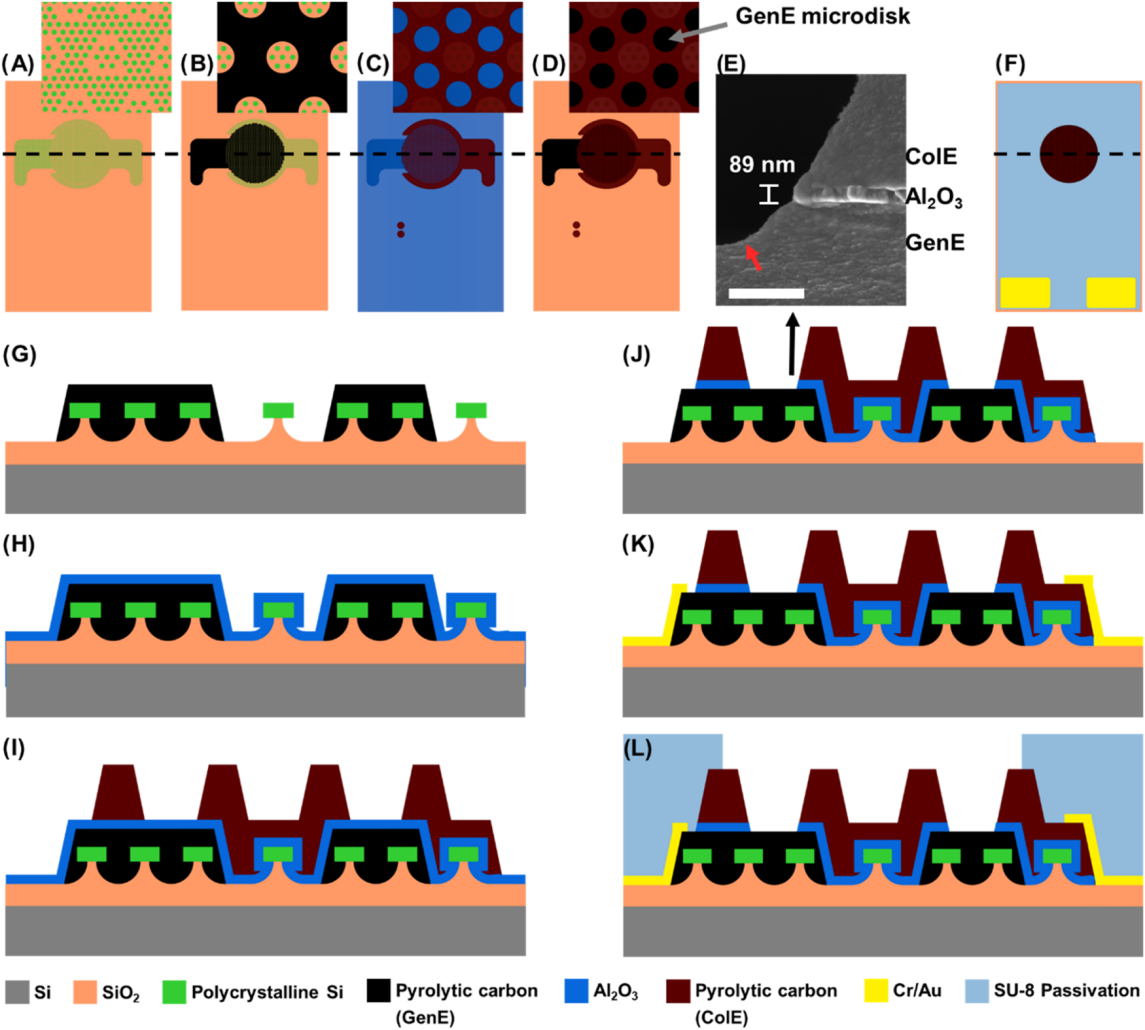
Design and microfabrication process of SLNE. Top view of SLNE design after (A) fabrication of adhesion structures, (B) maskless UV lithography of mr-DWL-photoresist precursor and pyrolysis to achieve bottom PyrC layer for the GenE, (C) ALD of Al_2_O_3_ followed by maskless UV lithography of mr-DWL-photoresist precursor and pyrolysis to achieve top PyrC layer for the ColE, and (D) RIE of Al_2_O_3_ realizing access with analyte on GenE microdisks. (E) SEM cross section of realized nanogap at the edge of the GenE microdisk. (F) Top view of design after electrical contact and passivation layer definition. Dashed lines in (A–D, F) correspond to cross-section schematic after (G) bottom PyrC layer fabrication, (H) ALD of Al_2_O_3_, (I) top PyrC layer fabrication, (J) RIE of Al_2_O_3_ realizing GenE microdisks, (K) metallization of electrical contacts, and (L) passivation layer maskless UV lithography.

The overall design of the PyrC SLNE consisted of a stack of three main layers, as illustrated in Figure 1: A bottom PyrC GenE, a top PyrC ColE, and an Al_2_O_3_ insulating spacer were placed between the two PyrC layers. A common challenge of PyrC is the delamination of electrodes during electrochemical measurements due to residual stress within the carbon generated in the fabrication process. Therefore, PolySi adhesion structures with a diameter of 8 μm (Figure 1A) hexagonally arrayed with a pitch of 14 μm were designed ensuring anchoring of GenE and ColE to the Si substrate by mechanical interlocking as demonstrated earlier.^48^ To enable the anchoring of the top ColE, microholes were implemented in the bottom GenE layer (Figure 1B). Additionally, large carbon contact pads with a size of 2.3 mm × 1.9 mm were included for both GenE and ColE providing an interface region with the metal contact layer with sufficient tolerance for aligning the shadow mask during metal deposition. After ALD of the insulating layer (Figure 1C), the hexagonal GenE microdisk array was defined by the geometry of the ColE (Figure 1D). The nanogap between GenE and ColE was situated at the perimeter of the GenE microdisks (Figure 1E) with the gap given by the thickness of the Al_2_O_3_ layer. Following metal deposition, a passivation layer defined the electrochemically active region with an area of 12.56 mm^2^ as well as the electrical contact area (Figure 1F).

#### 2.1.2 Fabrication of PyrC SLNE

**Figure 2:**
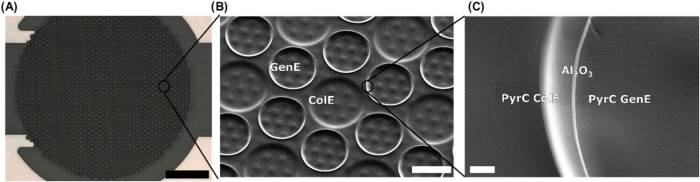
(A) Optical image of PyrC SLNE after completed microfabrication. (B) SEM image of GenE microdisks defined by ColE layer. (C) Edge of GenE microdisk with different layers defining the nanogap.

A novel fabrication process for the PyrC SLNE was developed as illustrated in Figure 1G–L. The GenE was fabricated through maskless UV photolithography of the mr-DWL 5 photoresist and subsequent pyrolysis at 1050 °C in inert atmosphere (Figure 1G). For the insulating spacer layer, several aspects regarding the material choice and deposition process were considered: (i) The bottom PyrC layer is nonplanar and has a thickness of 2.3 μm, with a high sidewall slope. Therefore, the deposition of the insulating material should be conformal (Figure 1H) and with a minimal number of defects ensuring proper electrical insulation of the two electrodes; (ii) the material should possess a high dielectric breakdown strength; (iii) the ColE was obtained with a second pyrolysis step of mr-DWL 5 photoresist patterned by UV photolithography (Figure 1I). Due to pyrolysis temperatures being ≥900 °C, thermal stability is a requirement for the spacer layer meaning it should not melt, decompose, or crack while it should act as a diffusion barrier for C atoms; (iv) the material must be mechanically stable since the photoresist precursor will shrink significantly during pyrolysis, imposing tensile stresses on the insulating layer^49^ and (v) it should adhere to PyrC, to prevent that the layers delaminate from each other, increasing the gap size. SiN, SiO_2_, and Al_2_O_3_ were considered as candidates due to their excellent mechanical, thermal, and breakdown strength.^32,41,50−52^ Reactive sputter deposition of SiN and SiO_2_ and ALD of Al_2_O_3_ were investigated. For SiN and SiO_2_, significant cracking and delamination of the films and the PyrC layers was observed (Figure S1A,B), while the layer stack after the Al_2_O_3_ process remained stable (Figure S1C). Thus, an ALD of 100 nm Al_2_O_3_ was selected for fabrication of the SLNE (Figure 1H). To prevent rupture of the Al_2_O_3_ layer due to potential outgassing or structural rearrangement of the bottom PyrC layer,^53^ the second pyrolysis step resulting in the PyrC ColE was performed at 900 °C (Figure 1I). This resulted in the Al_2_O_3_ layer shrinking to ∼89 nm thickness (Figure 1E) due to a phase change when annealed at high temperatures >800 °C, in which it densifies and becomes more resistant to chemical etching.^54^ The conformal deposition of Al_2_O_3_ on the substrate was confirmed by inspecting the adhesion structures, which provided mechanical interlocking with the carbon material even in the presence of the Al_2_O_3_ layer (Figure S2). Nanogaps between the two electrode terminals were achieved by chlorine-based reactive ion etching (RIE) of Al_2_O_3_ with the ColE acting as a self-aligning mask for the etching process (Figure 1E,J). The Al_2_O_3_ in the ColE microholes was completely etched revealing the GenE microdisks and enabling the analyte to access the bottom GenE. Here, it was observed that the etching sputtered away carbon material of the GenE (Figure 1E, red arrow), potentially increasing the diffusion zone overlap across the nanogap. Buffered Hydrofluoric acid wet etching was tested as an alternative to RIE to etch Al_2_O_3_ between the PyrC layers and increase the nanogap region. However, wet etching after pyrolysis was not feasible because the Al_2_O_3_ layer became chemically inert to HF^54^ (Figure S3A,B), while etching before the second pyrolysis process resulted in a short circuit between GenE and ColE (Figure S3C). To ensure good electrical connection with the external measurement setup, Cr/Au contacts were deposited on the carbon electrode contact pads by using e-beam evaporation (Figure 1K). Finally, a passivation layer of the SU-8 photoresist was deposited and patterned by photolithography to define the electrochemically active region of the SLNE (Figure 1L). Nanogap electrodes have thus been realized on wafer scale without the need for expensive techniques such as electron beam lithography and with a nanogap that can be adjusted solely by the thickness of the Al_2_O_3_ layer. An overview of the electroactive region of the SLNE and close-up views of the GenE microdisks is shown in Figure 2.

#### 2.1.3 Variation of GenE Microdisk Pitch and Radius

**Figure 3:**
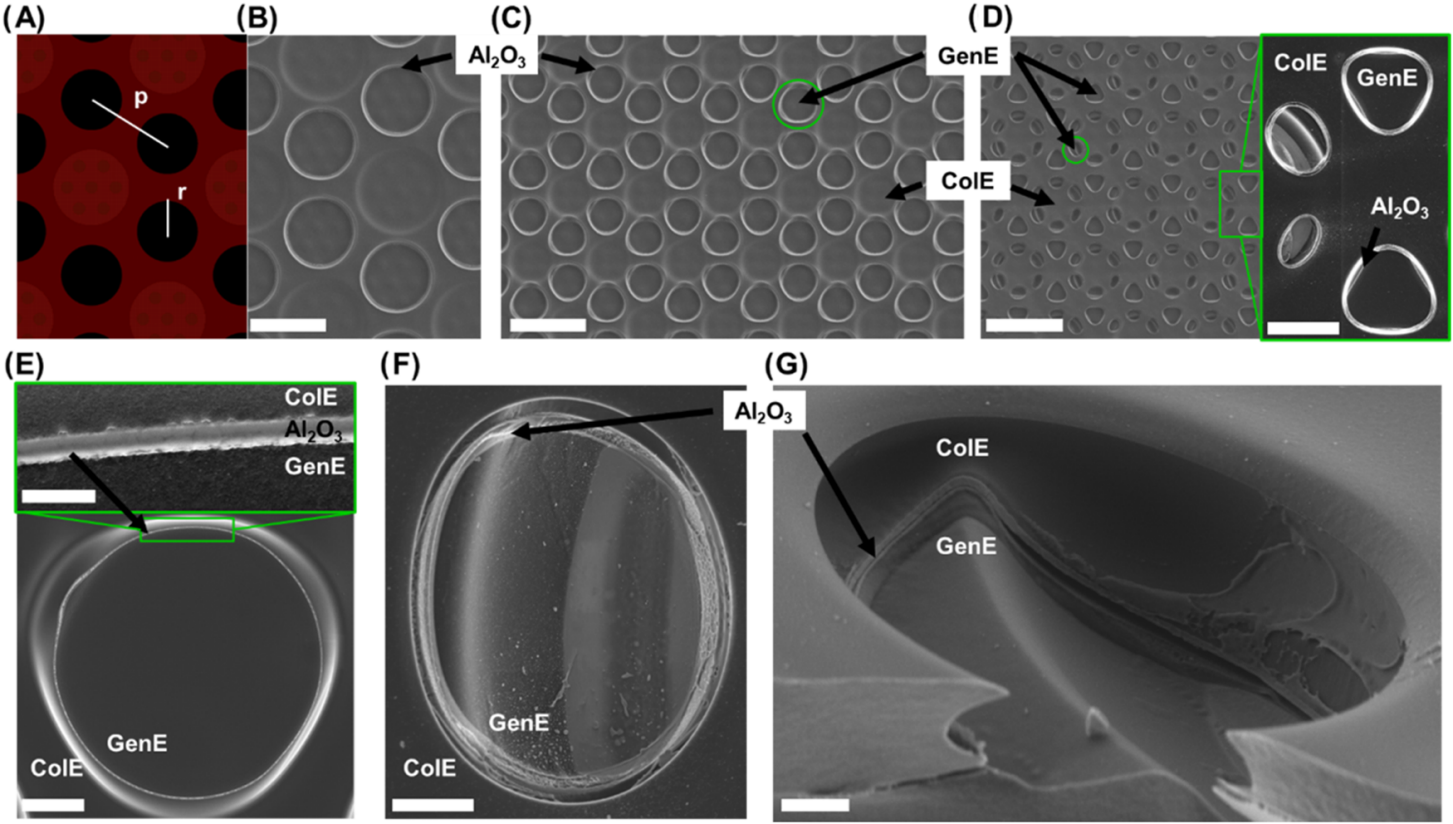
Overview of the three different SLNE designs. (A) Schematic of SLNE-L with definition of GenE microdisk pitch *p* and radius *r*. (B) Top-view SEM of SLNE-L. Scale bar = 50 μm. (C) Top-view SEM of SLNE-M. Scalebar = 50 μm. Green circles mark the close-up area in (E). (D) Top-view SEM of SLNE-S. The green circle marks the close-up area in (F). Scale bar = 50 μm, and for zoom-in, scale bar = 10 μm. (E) SEM close-up view of SLNE-M GenE microdisk with zoom-in of the region where the nanogap is defined by the Al_2_O_3_ layer. Scale bar = 5 μm, zoom-in scale bar = 500 nm. (F) SEM close-up view of the SLNE-S elliptical microdisk situated at an edge of the first PyrC layer. Scale bar = 2 μm. (G) Tilted close-up view of SLNE-S elliptical microdisk. Scale bar = 1 μm.

For Au-based SLNE, it has been demonstrated that decreasing the pitch of a microdisk array, while keeping the size of the disks constant improves electrochemical performance.^23,28^ Similarly, increasing the size of the microdisks, while keeping the pitch constant also increases the output current.^28^ Common for both reported studies is that the total area of the microdisks has not been kept constant, which is a parameter that also can affect the performance.^11^ In this study, two SLNE designs with identical total GenE area of ∼4.21 mm^2^ were fabricated while pitch *p* and radius *r* of the GenE microdisks were varied as described in Table 1. SEM images of the SLNE-L and SLNE-M designs after microfabrication are shown in Figure 3A–C. A third SLNE design was fabricated to further increase the total nanogap perimeter by pushing toward the resolution limit in the photolithography step used for the definition of the ColE photoresist precursor structures. In this SLNE-S design elliptical microdisks with an average semiminor axis of 3.2 μm and average semimajor axis of 4.1 μm were situated at a sidewall edge of the bottom PyrC layer with *p* = 14.0 μm (Figure 3D). The nonplanar topography of the elliptical microdisks might enhance the performance of nanogap electrodes by increasing the effective diffusion zone overlap region across the nanogap. Additionally, triangular microdisks with an area corresponding to a circle of *r* = 5.9 and *p* = 28.9 μm were placed on top of the flat region of the bottom PyrC layer (Figure 3D, zoom-in). The hexagonal structure of all of the designs was made to accommodate the stress induced by the shrinkage of the structures occurring during pyrolysis, to achieve mechanical anchoring of both GenE and ColE, as well as to minimize resistive losses in the PyrC electrodes. The particular heterogeneous geometry of SLNE-S, resulted from pushing the dimension of microdisks close to the resolution limit of this particular process. Close-up views of the individual microdisk structures are shown in Figure 3E–G. For the GenE microdisks of SLNE-M, the nanogap introduced by the Al_2_O_3_ layer circling the perimeter of the microdisks in plane is visible (Figure 3E). For SLNE-S, the nanogap perimeter conforms well to the nonplanar topography of the bottom PyrC layer (Figure 3F,G). Table 1 shows that while the total GenE area is smaller for SLNE-S, its estimated total GenE nanogap perimeter should be larger than those for the other two designs. It should be noted that for SLNE-S the ColE area with microdisks overlapped with the Si substrate region, where no PyrC was present, which meant that their electrically active area was effectively a factor of 0.7 compared to their overall area.

**Table 1:** Summary of the Geometrical Parameters for the Three Different PyrC SLNE Designs

design	GenE microdisk radius *r* [μm]	GenE microdisk pitch *p* [μm]	#GenE microdisks	total GenE area [mm^2^]	total GenE gap perimeter [mm]
SLNE-L	21.5	57.7	2900	4.2	392
SLNE-M	10.8	28.9	11,600	4.2	784
SLNE-S	elliptical: 3.2; 4.1	elliptical: 14.0	elliptical: 34,800	2.2	888
	circular: 5.9	circular: 28.9	circular: 11,600		

### 2.2 Effect of Redox Cycling Mode on Capacitive and Redox Current

#### 2.2.1 Electrical and Electrochemical Assessment of Electrode Separation

The electrical resistance between GenE and ColE after the fabrication was probed using a multimeter, and >80% of the SLNE displayed an interelectrode resistance of >2 MΩ which gradually increased over time similar to a capacitor, confirming that the two electrode terminals were electrically separated. This was further validated by applying a potential in a 2-electrode configuration with the ColE connected as counter electrode (CE) and reference electrode (RE) and the GenE as working electrode (WE) (Figure S4A) clearly showing a different response for SLNE with a short circuit compared to electrically separated electrodes. Lastly, electrochemical impedance spectra were recorded with both GenE and ColE connected as a single WE and with each of them being used individually (Figure S4B). Here, the sum of the charge transfer resistances for the individually applied GenE and ColE corresponded well to the resistance measured for both of them connected simultaneously. This confirmed the presence of a functional insulating layer between the GenE and the ColE of the PyrC SLNE. For further electrochemical studies, all SLNE were initially probed with a multimeter and the resistance measurements were used to assess electrode viability.

#### 2.2.2 Capacitive Background Current for Different CV Modes

**Figure 4:**
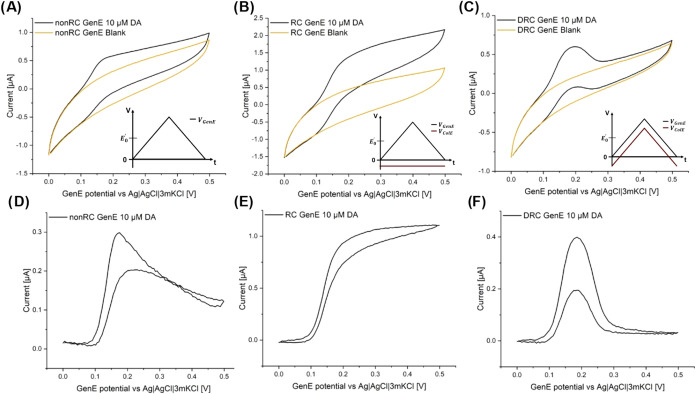
Comparison of capacitive background currents and redox currents for different CV modes. Current response in 1× PBS solution recorded for a GenE potential sweep from 0.0 to 0.5 V at 50 mV s^–1^ scan rate (orange lines) and 1× PBS with 10 μM DA (black lines) for different CV modes: (A) nonRC mode with ColE disconnected, (B) RC mode where a fixed −0.1 V potential is applied to ColE, and (C) DRC mode where ColE is swept along with GenE at an offset of −0.1 V. (D–F) The corresponding background-subtracted current signals. Measurements were performed using an Ag|AgCl|3MKCl RE and Pt wire CE, and the 5th scan is displayed. The insets in (A–C) show the potential applied vs time.

Significant capacitive background currents were observed during cyclic voltammetry (CV) with SLNE in PBS. Therefore, the background currents were characterized in detail for three different CV modes employing the SLNE-M: (i) “nonRC” mode, where the GenE potential was scanned while the ColE was disconnected, i.e., left floating identical as in standard CV (Figure 4A); (ii) “RC” mode, where a fixed potential bias was applied on the ColE while the GenE potential was scanned, which is the mode that is most often used for RC using CV (Figure 4B); (iii) “DRC” mode, where both GenE and ColE were scanned simultaneously with a fixed offset potential of −0.1 V (Figure 4C). The results show that the background current in PBS solution is highest for the RC mode and lowest for the DRC mode, where the latter is beneficial for electrochemical sensing. Furthermore, the background currents had a large time constant meaning that the current changed in the same time scale as for the CV. For all modes, the current variation was >1 μA from 0 to 0.5 V.

#### 2.2.3 Comparison of Current Response for Different Redox Cycling Modes

DA was used as a model analyte to assess the current response for the three different redox cycling modes with SLNE-M. Since DA can foul the electrode and thus affect subsequent electrochemical measurements, a low concentration of 10 μM was used. This in turn meant that the background current in PBS was comparable to the redox current signal from DA requiring its subtraction in DA measurements. The formal oxidation potential of DA was around 0.15 V. The current with DA present in the solution in its reduced form recorded in PBS below 0.1 V GenE potential coincided well with the background currents measured without DA (Figure 4A–C). This indicated that the sensor response was stable and that background signal subtraction was feasible (Figure 4D–F). Above 0.1 V GenE potential, the background-subtracted current response was different for the three modes. The RC mode resulted in the largest current response due to the presence of DA (Figure 4E). Notably, the DRC mode displayed an anodic current peak (Figure 4F) despite the RC because the potential sweep for both GenE and ColE ended at an oxidizing potential bias.

The sigmoidal shape in RC mode and the peak in DRC mode verify the successful fabrication of two electrically separated electrodes using Al_2_O_3_ as an insulating spacer layer. An explanation for the higher background current for nonRC and RC modes compared to DRC mode could be an increase in capacitive currents, since in an electrochemical capacitor, the non-Faradaic current increases with increasing potential difference between the two electrodes. A notable implication of stacking the electrode layers is that the electric field generated in one electrode will affect the other electrode.^23,55,56^ Thus, even in nonRC mode with the ColE left floating, the background current will also increase with potential due to charge build-up. This could explain the large background current even in the nonRC mode. On the one hand, a potential disadvantage of running the measurements in RC mode is that the SLNE should be calibrated to account for background currents, whereas the peak in DRC mode offers self-calibration. On the other hand, the current for RC mode was substantially larger than the peak current in DRC mode. This means that for the detection of low analyte concentrations, it is required to compare the signal-to-noise ratios of the systems.

### 2.3 Redox Cycling Performance of Different Nanogap Electrode Designs

#### 2.3.1 Describing the Performance in Redox Cycling Systems

To characterize RC performance, several parameters describing the system were investigated. Two types of amplification factors were considered inspired by the discussion by Wolfrum et al.^57^ The amplification factor AF, which is the one typically reported in the literature, is defined as the ratio between the anodic limiting current of the RC mode at 0.5 V and the anodic peak current of the nonRC mode
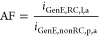
1where the subscripts GenE, RC, l, a, nonRC, and p represent generator electrode, redox cycling, limiting, anodic, nonredox cycling, and peak, respectively. AF describes the ability of the system to selectively amplify the current for a reversible compound. In this study, the anodic peak current was used as the limiting current in the nonRC mode in eq 1. The “amplification sensitivity factor” AF_sen_ is here defined as the ratio of limiting current density measured with the SLNE in RC mode divided by the anodic peak current density of macroelectrodes fabricated with the same electrode material (Figure S5)
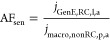
2where *j* is the current density. AF_sen_ attempts to compare the effect of structural and geometrical electrode parameters on signal amplification with electrode material contributions, i.e., trying to assess an eventual gain by switching from a large electrode to a micro- and nanostructured RC electrode design with the same material. It can also be used to determine if there is self-induced RC in the nonRC mode.^23^ In this study, AF_sen_ for the SLNE was calculated in two ways. First, based on the total footprint (FP) area of the electrochemically active region including both GenE and ColE results in AF_sen,FP_. Second, based on the GenE area resulting in AF_sen,GenE_ to estimate the amplification compared to using a macroelectrode with the same area as the GenE (Table 1). Thus, the current amplification is also compared to an electrode with a well-defined area, which can serve to elucidate the increase in performance across different designs. The collection efficiency η is defined as the ratio between the absolute value of limiting anodic and cathodic currents
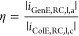
3where the subscript ColE represents collector electrode and c represents cathodic and describes the fraction of DA-Q that is reduced back to DA. A similar parameter can be used to compare the peak currents in DRC mode
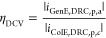
4where the subscript DRC represents differential redox cycling.

#### 2.3.2 Comparison of SLNE Designs in RC and nonRC Modes

**Figure 5:**
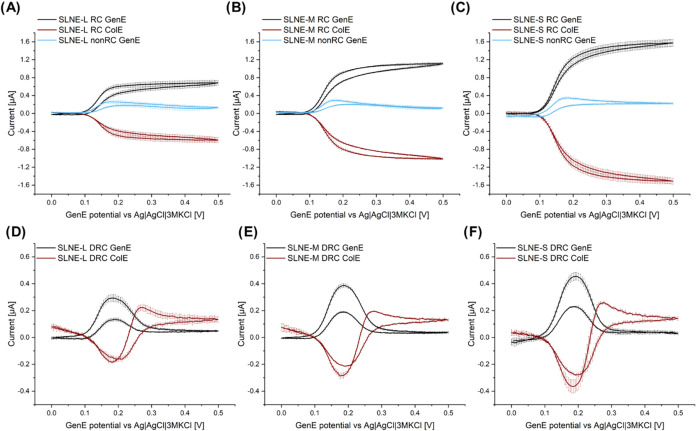
RC performance for different SLNE designs. Background-subtracted current vs GenE potential measured for 10 μM DA in 1× PBS solution where the GenE potential is swept from 0 to 0.5 V at a scan rate of 50 mV s^–1^ for (A–C) RC and nonRC modes and (D–F) DRC mode. (A, D) SLNE-L, (B, E) SLNE-M, and (C, F) SLNE-S. For RC mode, a fixed −0.1 V potential is applied to ColE, and for DRC, the ColE potential is swept at the same scan rate as the GenE potential at an offset of −0.1 V. Error bars denote the standard deviation across chips from the same wafer with *n* = 3. Measurements were performed using an Ag|AgCl|3MKCl RE and Pt wire CE, and the 5th scan is displayed.

Based on the analysis in Section 2.2, background current responses were subtracted for all DA measurements. The designs shown in Figure 3 were first compared using RC and nonRC modes to investigate the effect of different sizes of GenE microdisks on the current responses and to probe the effect of increasing the nanogap perimeter length. The results are shown in Figure 5A–C. The corresponding amplification factors, collection efficiencies, peak currents, and steady-state currents are summarized in Table 2.

**Table 2:** Electrochemical RC Performance Parameters for Different SLNE Designs and RC Modes

design	*i*_GenE,nonRC,p,a_ [μA]	*i*_GenE,RC,l,a_ [μA]	AF	*i*_nl_ [μA mm^–1^]	AF_sen,FP_	AF_sen,GenE_	η_RC_	*i*_GenE,DRC,p,a_ [μA]	η_DRC_
SLNE-L	0.23 ± 0.04	0.64 ± 0.04	2.8 ± 0.3	1.64	2.9	8.8	0.86 ± 0.02	0.27 ± 0.03	0.62 ± 0.02
SLNE-M	0.27 ± 0.04	1.03 ± 0.01	3.9 ± 0.6	1.32	4.7	14.1	0.92 ± 0.01	0.37 ± 0.02	0.71 ± 0.03
SLNE-MPP	0.18	0.69	3.9	1.36	3.1	18.5	0.90	0.09	0.67
SLNE-S	0.34 ± 0.03	1.46 ± 0.06	4.3 ± 0.5	1.64	6.7	38.3	0.94 ± 0.02	0.43 ± 0.03	0.80 ± 0.05

Generally, SLNE designs with a smaller microdisk size yielded higher currents in both RC and nonRC modes. However, the behavior of the nonRC current differed for SLNE-S compared to the other designs by having a more normal CV-like response with a clear oxidation peak followed by a decreasing current in the oxidizing regime. For SLNE-L and SLNE-M, a peak also occurred, but the limiting current behavior was different. The current decreased during the forward scan after the oxidation peak and to a larger degree than for standard diffusion-limited behavior observed for SLNE-S. In the backward scan, the current increased until the formal potential of DA at ∼0.15 V was reached. This unusual limiting current behavior is explained by a similar reasoning as it was discussed for RC mode in,^17^ attributing it to the close proximity of the GenE and ColE affecting the actual potential experienced by the analyte. In the nonRC mode, the cathodic peak on the backward scan was close to nonexistent for all designs as expected since DA is only quasi-reversible.^19^ For the RC mode, both GenE anodic and ColE cathodic currents reached a plateau for all PyrC SLNE designs. Furthermore, AF_sen,FP_ < AF_sen,GenE_ due to the limited area of the GenE indicating the importance of reporting the current amplification relative to a nonstructured electrode.

Since current increased with decreasing GenE microdisk diameters, while the total GenE area remained constant or even decreased (Table 1), it is here proposed that the main contribution to the higher currents is related to the nanogaps. As shown in Table 1, the total nanogap perimeter for the three-electrode designs increases by decreasing the microdisk diameters, which might enable a higher RC amplification. In fact, AF increased from 2.8 for SLNE-L to 4.3 for SLNE-S, meaning that it increased as the nanogap perimeter increased. The current amplification AF_sen,GenE_ increased from 8.8 using SLNE-L to 38.3 for SLNE-S. Since AF_sen,GenE_ directly compares the current amplification from GenE in RC mode to a macroelectrode with a well-defined area, this provides an explanation that the amplification is improved as the nanogap perimeter is increased (Table 1). However, Fu et al.^23^ also reported that the current increased with a higher density of GenE microdisks and attributed this to increasing diffusion zone overlap and secondary analyte trapping. To further explore these hypotheses, the total projected perimeter of the nanogaps (Table 1) was used to determine the current per nanogap length, *i*_nl_ (Table 2). Notably, the relative variation of this parameter was smaller compared to current per GenE area indicating that it is in fact the nanogap that provides the main contribution to current. Moreover, *i*_nl_ is slightly higher for SLNE-S and SLNE-L compared to SLNE-M. For SLNE-S this is attributed to the increased perimeter due to nanogaps along the nonplanar topography. In the case of SLNE-L, it could be explained by the more isolated nanogap perimeter due to the larger feature size and, in turn, better diffusion profiles along the nanogap. Additionally, the low variation in the current response for the different designs provides evidence that the performance of the chips was reproducible.

#### 2.3.3 Comparison of SLNE Designs in DRC Mode

The DRC peak currents shown in Figure 5D–F were higher than nonRC peak currents but several times lower than those for the RC mode. The collection efficiency of DRC mode also tended to lower values compared to RC mode. These observations could be explained by the lower potential difference between the GenE and ColE when using DRC mode compared to the RC mode. In fact, the peak currents in the DRC decreased when the potential offset was decreased (Figure S6). For the DRC mode, a peak can also be seen in the ColE current when the GenE potential is at ∼0.25 V and the ColE potential correspondingly at ∼0.15 V due to the oxidation of DA that is present in the vicinity of the electrode. Afterward, the ColE current is attaining a diffusion-limited current while “shielding” the oxidation of DA at the GenE. Also, on the return scan the cathodic peak at the ColE shifted to a more positive value than for the GenE. This is attributed to additional DA-Q being present enhancing the diffusion rate before maximum RC rate is reached.

### 2.4 Partial Passivation of SLNE

**Figure 6:**
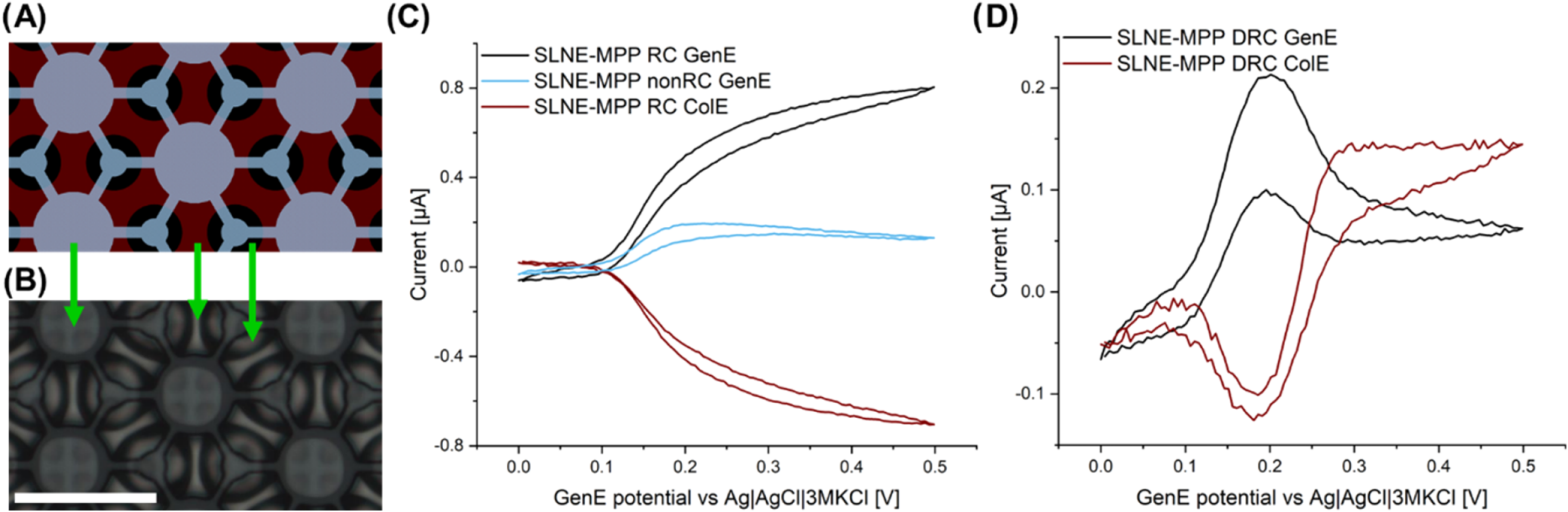
SLNE with partial passivation. (A) Schematic and (B) optical image of a small section of the electrochemically active region in SLNE-MPP with partial passivation (gray area in (A)). Green arrows indicate the layers from the schematic at the corresponding points in the optical image. (C, D) Background-subtracted current vs GenE potential for 10 μM DA in 1× PBS solution where the GenE potential is swept from 0 to 0.5 V with at a scan rate of 50 mV s^–1^ for (C) RC and nonRC modes and (D) DRC mode. Measurements were performed using an Ag|AgCl|3MKCl RE and Pt wire CE instrument, and the 5th scan is displayed.

To investigate if RC performance could be improved by confining electron transfer to the vicinity of the nanogaps and reducing contributions of the GenE and ColE areas that are not in its immediate proximity, a partial passivation using the SU-8 photoresist was defined on the electrochemically active region (Figure 6A,B). Due to the well-defined planar areas of the SLNE-M, this design was modified. The partial passivation design SLNE-MPP consisted of concentric circles with a diameter of 10 μm covering the centers of the GenE microdisks, larger circles with a diameter of 29 μm on the ColE in between the GenE microdisks and connections between the two circular shapes by a 4 μm wide line to reduce the risk of delamination. This resulted in a decrease in surface area of ∼51% for GenE and ∼46% for ColE, while the nanogap perimeter decreased by 36%. The RC parameters for SLNE-MPP are summarized in Table 2. The current response of SLNE-MPP for RC, non-RC, and DRC modes was similar to SLNE-M, albeit slightly lower (Figure 6C,D). The decreased area resulted in a decreased current, but in a higher current density, with an AF_sen,GenE_ that was slightly higher than for SLNE-M. η was lower compared to the original design which could be explained by the significantly smaller size of the ColE. These results indicate that the main current contribution in RC and DRC mode originated from the proximity of the nanogap.

### 2.5 Dopamine Detection with Differential Chronoamperometry

Preliminary experiments employing the SLNE and standard CV for measurements of low concentrations of DA yielded a minimum detectable concentration of ∼1 μM. Here, the SLNE-M design was used for further investigation and optimization of DA detection with the two different RC modes with the aim to improve the minimum detectable concentration. For this purpose, several aspects were considered: (i) For RC mode, the limiting current is determined by considering the entire voltage range from 0 to 0.5 V, which for DRC is reduced to 0.1 to 0.2 V. Therefore, the background current drift over the voltage range used was larger in RC mode compared to DRC mode even in cases where the overall noise was larger for DRC (Figure S7); (ii) the current amplitude was lower in DRC compared to RC, which in part was because no steady-state was achieved in DRC mode.

**Figure 7:**
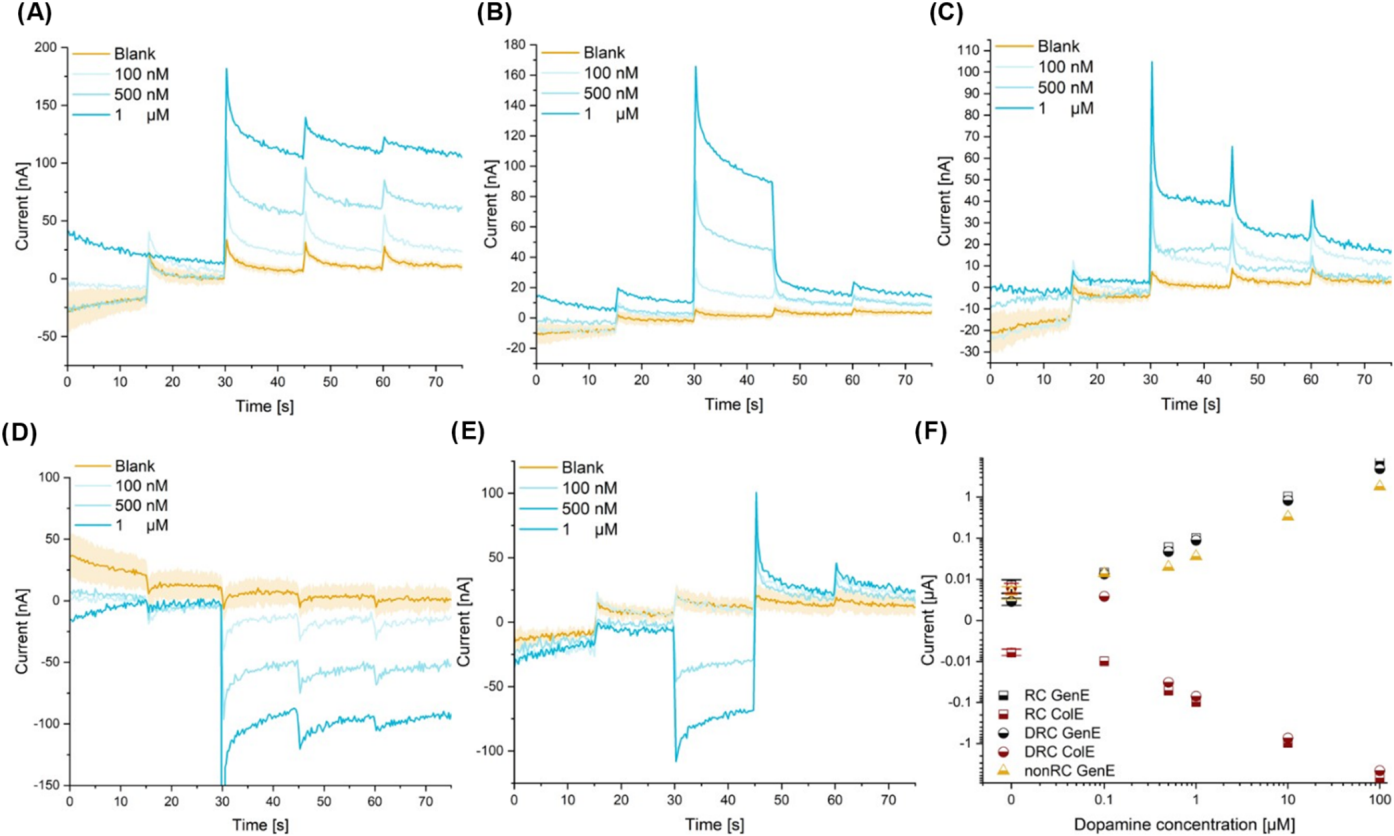
Electrochemical detection of DA with SLNE-M. Five-step CA with varying concentrations of DA for (A) RC mode GenE, (B) DRC mode GenE, (C) nonRC mode GenE, (D) RC mode ColE, and (E) DRC mode ColE. For RC mode measurements, the ColE potential was fixed at −0.1 V; for DRC, it was stepped together with the GenE potential at an offset of −0.1 V and disconnected for nonRC. For all modes, the GenE potential was stepped from 0 to 0.4 V in steps of 0.1 V with a dwell time of 15 s at each step. The shaded region of the curve for blanks corresponds to the standard deviation for separate blank measurements by using the same chip. *N* = 3. (F) Calibration curve for DA in 1× PBS for GenE and ColE. Measurements were performed using an Ag|AgCl|3MKCl RE and Pt wire CE.

To increase the current output of DRC mode while narrowing the voltage range used to measure the RC current, multistep chronoamperometry (CA) in conjunction with the different RC modes was proposed and investigated. The GenE potential was stepped from 0 to 0.4 V in steps of 0.1 V, each with a duration of 15 s. For RC mode, the ColE was biased at −0.1 V for all steps, whereas it in DRC mode followed the stepping of the GenE potential at an offset of −0.1 V. This novel hybrid approach combining CA with DCV, here termed differential chronoamperometry (DCA), reduced the voltage range for current estimation to 0.1 V for all modes, meanwhile allowing the DRC mode to reach a steady state. A simplified schematic of the voltage vs time is shown in Figure S8. The current response of the 5-step CA for all RC modes is shown in Figure 7, and the measurements for higher concentrations are displayed in Figure S9. The response for the RC mode is characterized by a large current step at 0.2 V reaching a plateau maintained even for subsequent increases in the voltage. The DRC displayed a large current step from 0.1 to 0.2 V while the current decreased almost back to the baseline level at 0.3 V resulting in a “peaklike” overall response. For the nonRC mode, a drastic increase in current was also observed at 0.2 V which decreased for subsequent steps, although not as fast as that for the DRC mode.

The relative current response due to DA oxidation for each mode was determined by subtracting the average current at GenE = 0.1 V from the average current at GenE = 0.2 V. To avoid spikes after potential stepping, the current was averaged for the central 13 s at each step. The calibration curve resulting from the current measurements is shown in Figure 7F. The current response for all modes was linear with concentration between 100 nM and 10 μM. Notably, the sensitivity for the GenE current response of the DRC mode was approximately 80% of the one of the RC mode, which is a considerably larger fraction than for CV measurements. The sensitivities are lower than what has been reported for DA measurements using PyrC electrodes without RC.^2,58^ However, the values are higher than what has been achieved using RC with metal and PyrC electrodes,^12,59^ although it should be noted that in the latter case, the sensitivity was determined in a combined DA and ascorbic acid solution. The LOD was calculated as LOD = 3σ*a*^–1^, where *a* is the slope of the linear fit of the calibration curve and σ is the standard deviation of the blank measurements (Table 3). Although the highest sensitivity was achieved for RC GenE, DCA GenE demonstrated the lowest LOD, which can be explained by lower background currents. Interestingly, the LOD was better for RC ColE than DCA ColE, which might be due to the fixed potential of the ColE in RC mode. nonRC GenE showed the lowest sensitivity and highest LOD compared to RC and DCA modes.

**Table 3:** Sensitivity Determined from the Slope of Linear Fit of the Calibration Curve between 100 nM and 10 μM for the Different CV Modes^a^

	RC GenE	RC ColE	nonRC GenE	DCA GenE	DCA ColE
sensitivity [nA μM^*–*1^]	105	–97	33	83	–73
LOD [nM]	74	33	157	21	71

a The *R*^2^ was >0.999 for all modes. The standard deviation used to determine LOD was based on blank measurements using the same chip where the blank was a nitrogen-bubbled 1X PBS solution and *n* = 3.

**Figure 8:**
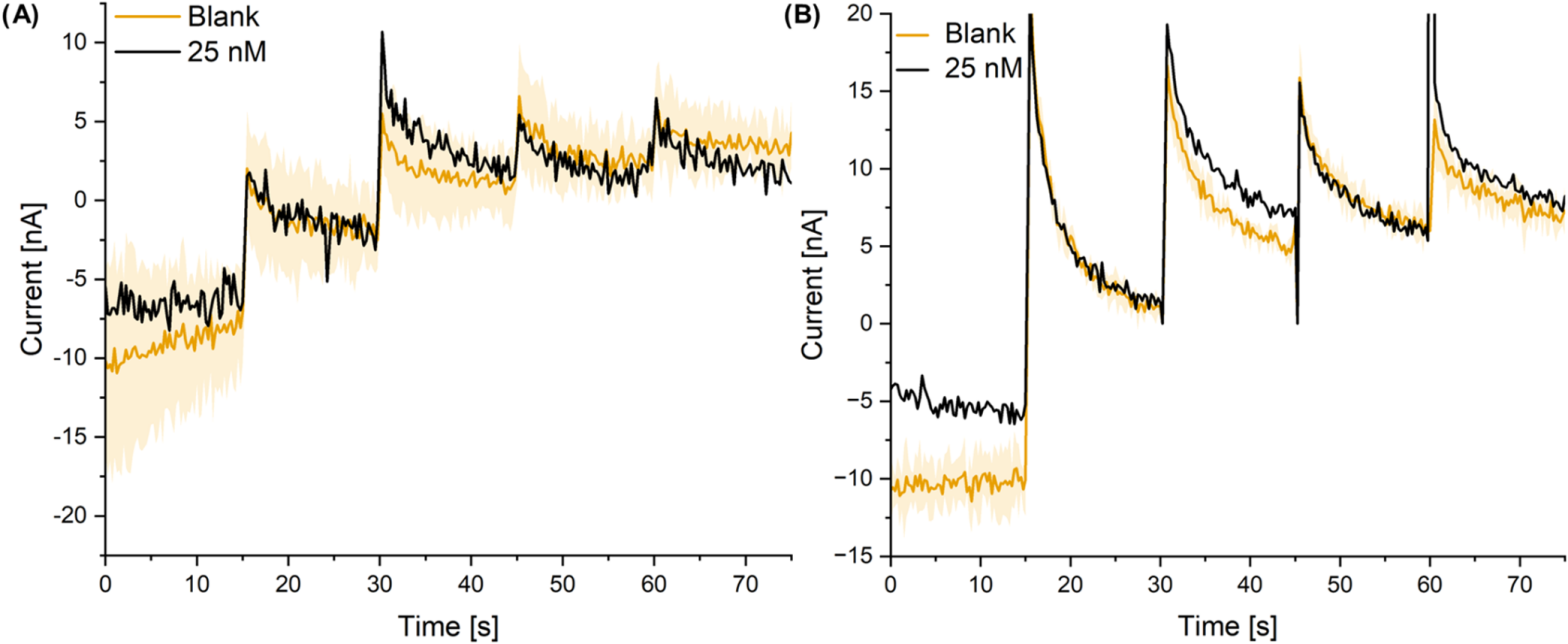
Detection of 25 nM DA with (A) SLNE-M and (B) SLNE-S using DCA. The curve in (A) corresponding to 25 nM DA has been shifted along the *y*-axis to match the blank curve in the region 15–30 s. The shaded region of the curve for blanks corresponds to the standard deviation for multiple separate blank measurements using the same chip. *N* = 3. Measurements were performed using an Ag|AgCl|3MKCl RE and Pt wire CE.

Finally, the LOD for GenE in DRC mode was challenged by performing the five-step DCA for the detection of DA at concentrations of 25 nM. Figure 8 shows that there is a distinct response with 25 nM DA added. For SLNE-M, the response lied within the shaded region corresponding to the standard deviation of the blank measurements, whereas for SLNE-S the recorded signal was clearly outside the shaded region. To test whether the responses were significant, a one-sided one-sample *t* test was conducted using the current step response for the 25 nM DA measurement as the population mean. The test showed that the p-value of the blank response was 0.019 and 0.0097 for SLNE-M and SLNE-S, respectively. Thus, the differences for both designs were statistically significant at a 5% level, but for the SLNE-S design even at a 1% level. Including the 25 nM measurement for the linear fit in the DCA measurements for SLNE-M did not change the sensitivity. Thus, for DCA GenE current, the linear range is 25 nM to 10 μM.

## 3 Discussion

To achieve high RC amplification, the interelectrode distance between two electrically separated electrodes should ideally be <100 nm.^60^ Additionally, for SLNE, a larger sensor area improves amplification^11,61^ which is often neglected. Here, a novel fabrication process for a PyrC-based SLNE was demonstrated. The SLNE was realized by stacking two PyrC electrodes separated by an insulating spacer of Al_2_O_3_ deposited by ALD. Nanogaps were realized by RIE of the Al_2_O_3_ layer achieving GenE PyrC microdisks with an 89 nm gap to a PyrC ColE. Thus, for the first time, nanometer gaps were realized on a PyrC-based platform using conventional cleanroom processing allowing the fabrication of large arrays of nanogaps in parallel. Even compared to metal-based SLNE redox cycling platforms, this is among the thinnest insulating spacers demonstrated, with Hüske et al. and Fu et al. demonstrating 100 nm and 50 insulating spacer thicknesses, respectively.^11,28^ For PyrC-based RC platforms, interelectrode gaps of 0.85 μm for interdigitated electrodes were demonstrated by Kamath et al.^62^ and 2.1 μm for a suspended mesh electrode.^63^ The interelectrode gap achieved here is thus an order of magnitude smaller. Furthermore, the process presented in this paper allowed for precise control of both lateral geometry and interelectrode gap size.

Another important aspect is the background noise and drift associated with the electrodes. Low background currents are crucial for the detection of low concentrations of analyte. Several other sources report noise levels in the fA to pA range.^13,64^ Meanwhile, drift can inhibit the use of electrodes in sensing or monitoring applications. In our study, the noise of the SLNE was in the 1 nA range, while the current drift was in the 1 μA range before and 1–10 nA range after background subtraction depending on the mode used. Some of the background signals could be due to capacitive coupling of the electrodes, which could possibly be improved by the increased layer thickness of the insulating layer in non-nanogap regions.

Different SLNE geometries were compared in terms of the RC current and amplification. The current magnitude and amplification increased as the disc size and spacing decreased. This finding conforms to Fu et al. and Han et al. which both reported an increase in amplification with decreasing pitch and constant disk or hole size.^23,28^ In addition, Han et al. reported that the RC current was proportional to the number of holes, i.e., the GenE surface area. In this study, our results strongly suggest that the current was proportional to the nanogap perimeter and that the amplification factor increased with an increasing nanogap perimeter. This was further confirmed with the partially passivated design, in which the RC current per nanogap perimeter remained approximately constant. Furthermore, Atighilorestani et al. considered recessed electrodes with varying heights and radii. They showed that the current density increased as the electrode height decreased.^65^ They also found that the largest contribution to current was due to the interelectrode gap and radius and not the height. Thus, taking into account the results from,^11,23,28,65^ we propose that increases in RC current for SLNE also stem from increased nanogap perimeter, in addition to the recapture effect by overlapping diffusion zones and nanogap size. Typically, the nanogap perimeter has not been estimated in the literature. Given the dimensions of the number of microdisks, or equivalently, holes and their radii, the perimeter is estimated to be ∼48 and ∼141 mm for refs (11,28), respectively. Meanwhile, Hüske et al.^61^ have reported SLNE with a nanogap perimeter estimated to be on the order of 10^5^ mm. In this study, the longest perimeter demonstrated was 888 mm which is an order of magnitude lower than Hüske et al., however, having a smaller nanogap of <100 nm compared to ∼400 nm demonstrated in their work.

One of the promising aspects of RC is the possibility to perform measurements in the DRC mode. Several authors have displayed its usefulness in terms of differentiating solutions with multiple reversible species.^43−45^ Here, we compared the DRC mode to the RC mode CV in terms of η and current magnitude and found that for the DRC mode, values were lower. This is ascribed to a smaller effective potential window. However, the background current for the DRC mode was also lower compared to the RC mode. Thus, we combined DRC mode and CA and proposed a novel technique which we coined DCA, providing improved RC performance, while maintaining the qualitative peaklike nature of DCV. This resulted in an improved LOD of 21 nm for DCA compared to 74 nM for RC mode CA using the GenE. The sensitivity of DCA however, was slightly lower at 83 nA μM^–1^ compared to 105 nA μM^–1^ for RC mode CA. These sensitivities and LOD are, to the best of our knowledge, state-of-the-art for DA RC. Previous studies have reported LOD and sensitivity values for DA RC of 200 nM and 0.63 nA μM^–1^ in the presence of 1 mM ascorbic acid^12^ and 1.08 μM and 0.042 nA μM^–1^ in artificial cerebrospinal fluid.^66^ Authors have also reported other nonRC-based methods for DA detection such as fast-scan cyclic voltammetry (FSCV) with an LOD of 3.7 nM and a sensitivity of 12.7 nA μM^–1^,^47^ as well as CA with an LOD of 0.025 nM and a sensitivity of 13.2 nA μM^–1^.^64^ Although these nonRC-based techniques have displayed better LOD, the sensitivity has been significantly lower than that reported here. Additionally, the non-RC-based techniques also typically require some sort of surface modification, but the electrodes used in our work were pristine samples.

The results presented in this study bring forward several considerations related to the fabrication of SLNE and the electrochemical technique employed for RC. Many nanogap electrodes reported in the literature have been fabricated using thin planar metal films deposited with e-beam evaporation,^13,23,67^ which inherently limits the ability to fabricate 3D structures. Several specific methods have been developed that might alleviate the challenges.^68,69^ However, the freedom to vary the geometry is typically limited.

The photoresist-derived PyrC material offers substantial freedom of design in electrode fabrication^12,35^ while the nanogaps reported here can potentially be integrated with more complex geometries. With additional optimization of the etching process, nanocavities could be realized, which could further enhance current signals while achieving a fast temporal coupling between bulk solution and nanogaps.^57^ Meanwhile this would allow the realization of large interface areas of nanogaps even with the use of standard UV lithography. Further increase of the signals given a specific footprint of the device is important for the more widespread adoption of RC systems. This has to be pursued keeping in mind the other options, such as the use of carbon nanotube electrodes, which have already demonstrated superior detection limits for, e.g., DA.^70^ Thus, it is important to also report other metrics of the RC performance than only the AF, such as the *AF*_*Sen*_ reported here and in other forms elsewhere.^23,61^

## 4 Conclusions

A fabrication process for a PyrC-based SLNE with large arrays of electrodes was demonstrated for the first time with nanogaps of 89 nm. The process facilitated variation of pitch and geometry of the microdisks, which were compared across several electrochemical RC modes of operation. DA was used as a model analyte, demonstrating amplification factors of up to ∼38. Finally, the detection limit for DA using SLNE was improved using DCA as a novel electrochemical technique where an LOD of 21 nM, a sensitivity of 83 nA μM^–1^, and a linear range from 25 nM to 10 μM were demonstrated. DA detection at a low concentration of 25 nM was shown experimentally, where the current response for spiked samples was determined to be significantly different from blank samples at a 1% level. Furthermore, the novel fabrication process provides an interesting perspective in realizing 3D nanogap electrodes using photoresist-derived pyrolytic carbon.

## 5 Experimental Methods

### 5.1 Microfabrication of Stacked-Layer Nanogap Electrodes

#### 5.1.1 Substrate Preparation and Adhesion Structure Fabrication

To ensure stability of pyrolytic carbon electrodes on Si-based carrier substrates, polycrystalline Si (polySi) adhesion structures were initially fabricated similarly as described earlier.^48^ Briefly, 6 in. Si wafers (Siegert Wafer GmbH, Germany) were oxidized to grow a 1 μm thick SiO_2_ followed by low-pressure chemical vapor deposition of 435 nm polySi. The adhesion structures were patterned by using a combination of maskless UV lithography and wet chemical etching to etch the polySi and SiO_2_ layers. Immediately after etching the SiO_2_ layer, the substrates were submerged into 98% H_2_SO_4_ at 80 °C with an addition of (NH_4_)_2_S_2_O_8_ for 10 min to clean the samples. The wafers were then rinsed in DI water and transferred to a 120 °C hot plate for 2 min to evaporate any residual water molecules. After baking, the wafers were placed on a solid metal chuck to cool them to room temperature by convection for approximately 1 min.

#### 5.1.2 Maskless UV Photolithography of Precursor Structures for GenE

Immediately after cooling, the substrates were spin-coated with a layer of mr-DWL 5 (microresist technology GmbH, Germany) using an RCD8 T spin coater (Süss MicroTec, Germany). The resist was manually deposited using static dispensing followed by ramping the spin speed at 500 rpm s^–1^ to 1150 rpm for 60 s to define a 9 μm thick mr-DWL layer. Then, the layer was soft baked on a hot plate (Harry Gestigkeit GmbH, Germany) at 50 °C for 15 min using a ramp of 2 °C min^–1^ followed by cooling by convection for 90 min. The mr-DWL film was exposed using maskless UV photolithography at a wavelength of 405 nm, postexposure-baked, developed, and hard-baked similarly as reported previously using a defocus value of 2 instead of 1.^48^

#### 5.1.3 Pyrolysis of mr-DWL and Atomic Layer Deposition of Al_2_O_3_

The mr-DWL film was subjected to a 6-step pyrolysis scheme described previously^48^ with a maximum dwell temperature of 1050 °C resulting in the first PyrC layer defining the GenE with a thickness of 2.3 μm. The wafers were immediately subjected to ALD of Al_2_O_3_ using a Picosun thermal ALD model R200 (Picosun Oy, Finland). The process consisted of 1000 cycles alternating between 0.1 s pulses of H_2_O and tetramethylaluminum (TMA) at 300 °C under 200 and 150 sccm N_2_ flow rate, respectively, resulting in a conformal 100 nm thin Al_2_O_3_ layer.

#### 5.1.4 Maskless UV Photolithography and Pyrolysis for ColE

The substrates then underwent the same mr-DWL photolithography process as described above one more time to define the precursor structures for the ColE. After hard baking, the wafers were exposed to a plasma descum at 1000 W for 100 s with flow rates of 400 and 70 mL min^–1^ for the O_2_ and N_2_ gases, respectively, to remove residual photoresist scum layers. A similar 6-step pyrolysis process was then employed but with a maximum temperature of 900 °C to convert the photoresist precursor structure into the PyrC ColE.

#### 5.1.5 Reactive Ion Etching of Al_2_O_3_ Realizing Nanogap Electrodes

To prevent physical sputtering of the remaining SiO_2_ layer across the wafer, the substrates were masked with a photoresist during RIE of the Al_2_O_3_ layer. The substrates were pretreated with Hexamethyl Disilazane (HMDS) before spin-coating (Süss MicroTec Gamma 4M spin coater, Germany) of a layer of 4 μm thick AZ MiR photoresist (MicroChemicals GmbH, Germany). The film was exposed using maskless UV photolithography on an MLA150 (Heidelberg Instruments, Germany) equipped with a 375 nm laser with a dose of 750 mJ cm^–2^ and a defocus of 0. After exposure, the film was postexposure-baked at 110 °C for 60 s and puddle developed for 120 s in 2.38% tetramethylammoniumhydroxide (TMAH, AZ 726 MIF Developer, Microchemicals GmbH, Germany) for 120 s (Süss MicroTec Gamma 2M developer). The wafers were then treated with a brief O_2_ RIE process for 60 s with 10 sccm oxygen gas flow, 120 W coil power, and 15 W platen power at 10 mTorr in a PRO ICP (SPTS Technologies Ltd., England). After this, they processed for 15 min in BCl_3_- and Cl_2_-based plasma with flow rates of 40 and 15 sccm, respectively, at a coil power of 500 W and platen bias of 70 W at 3 mTorr. Both processes were performed at 0 °C. This completely removed the Al_2_O_3_ on the GenE, resulting in 89 nm nanogaps between PyrC microdisk GenE and PyrC ColE. The photoresist mask was then removed using PGMEA (mr-Dev 600, micro resist technology GmbH, Germany).

#### 5.1.6 Definition of Metal Contacts

After being etched, metal contacts were defined by e-beam evaporation (Temescal, Ferrotec) through a shadow mask. The shadow mask was prepared using microSTRUCT vario laser machining equipment (3D-Micromac AG, Germany) on a 150 μm thick steel sheet. The sheet was clamped to the wafers with magnets and deposition of a layer of 20 nm Cr at a deposition rate of 2 Å s^–1^ followed by 200 nm Au at a deposition rate of 5 Å s^–1^ at a vacuum level of at least 10^–6^ mbar was performed.

#### 5.1.7 Photolithography of Passivation Layer

The wafers were treated with a cleaning process to remove potential AlCl etch residuals. The wafers were rinsed three times in a beaker with fresh DI water and then for 3 min in a beaker with ethanol after which they were left to dry in a fume hood. They were then transferred to a furnace, in which they were baked at 200 °C for 2 h at a vacuum of 2 mbar. The passivation layer was defined as described earlier,^48^ but with an exposure dose of 1000 mJ cm^–2^ and a defocus of 2.

#### 5.1.8 Dicing of Wafer

Finally, the wafers were diced using a DAD 3421 Automatic Dicing Saw (Disco, Japan) followed by megasonic cleaning of the chips (Wafer Cleaner DCS1441, Disco, Japan). This resulted in 82 chips from each 6-in. wafer.

### 5.2 Electrochemical Characterization

To evaluate the performance of the different RC modes and assess the potential of the SLNE as sensors, electrochemical measurements with dopamine were performed. All measurements were conducted in a 10 mL glass beaker with 4 mL of solution using a custom-made 3D printed holder (Figure S10), with an external Pt CE and Ag|AgCl|3MKCl RE. All DA CV measurements were performed using 10 μM DA in a nitrogen-bubbled 1× PBS solution with 137 mM NaCl, 2.7 mM KCl, 10 mM Na_2_HPO_4_, 1.8 mM KH_2_PO_4_, using a scan rate of 50 mV s^–1^ unless otherwise stated. The measurements were performed using a PalmSens4 potentiostat with a bipotentiostat module included and using the PSTrace 5.9 software. The top PyrC was always used as the ColE and the bottom as the GenE. To stabilize background current drift using CV, 20–30 scans were performed depending on the SLNE and RC mode until the drift between scans was <10 nA at the relevant voltage, which e.g., for RC mode was 0.5 V. Then, 5 scans were performed in PBS solution freshly bubbled with nitrogen two times before a measurement. To stabilize background drift for CA measurements, five 5-step CA measurements were initially performed in freshly nitrogen-bubbled PBS. Subsequently, the solution was replaced with another PBS solution and a 5-step CA was performed, with this procedure performed twice between measurements with DA and once for blanks.

## Supporting Information Available

Additional experimental details including results from supporting electrochemical experiments; optical micrographs of structures during process development; DCA potential vs time scheme; and photograph of the experimental setup (PDF)
