# Supplementary material for: Electrochemical Redox Cycling with Pyrolytic Carbon Stacked-Layer Nanogap Electrodes
Source: ACS Appl Mater Interfaces. 2025 Feb 19;17(9):14375–88. doi: 10.1021/acsami.4c18998 (PMC11892468; doi:10.1021/acsami.4c18998)
Supplement: Supplementary file 1 — am4c18998_si_001.pdf [file am4c18998_si_001.pdf]

Supporting Information

Electrochemical Redox Cycling with Pyrolytic Carbon Stacked Layer Nanogap Electrodes

Nicolai Støvring[a]*, Arto R Heiskanen[b], Jenny Emneus[b], Stephan Sylvest Keller[a]

[a] National Centre for Nano Fabrication and Characterization, DTU Nanolab, Technical University of Denmark, Kgs. Lyngby, 2800, Denmark
[b] Department of Biotechnology and Biomedicine, DTU Bioengineering, Technical University of Denmark, Kgs. Lyngby, 2800, Denmark

*Corresponding author: Nicolai Støvring; E-mail: nicosto@dtu.dk

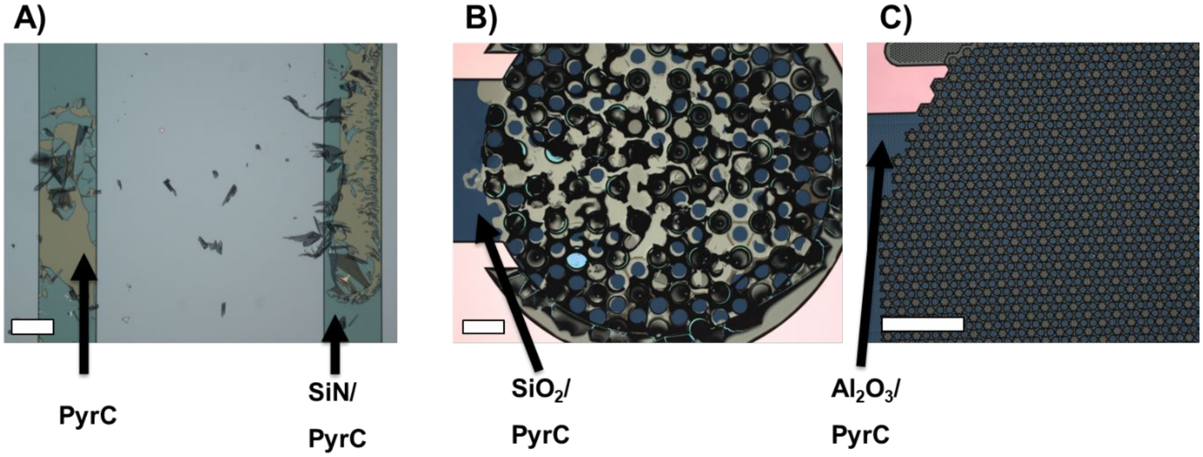

Figure S1: Cracks and delamination using reactive sputter deposition of A) SiN after pyrolysis of the second photoresist layer for the ColE. B) SiO_2 after second pyrolysis. C) absence of cracks and delamination using ALD Al_2O_3 process after second pyrolysis. Scalebars= 500 µm for all images.

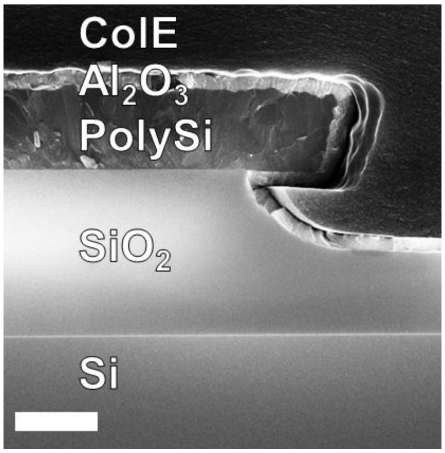

Figure S2: Adhesion structure after ALD of Al_2O_3 and second pyrolysis step showing mechanical interlocking with the PyrC of the ColE layer. Scalebar = 500 nm.

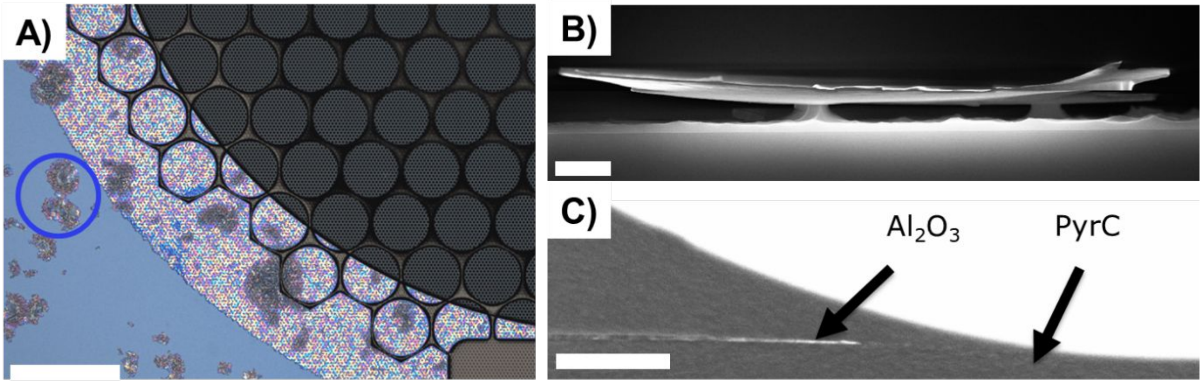

Figure S3: A) Optical image after wet etching for >10 min in BHF integrating the etching step after the second pyrolysis step in which the Al_2O_3 could not be etched. The blue circle marks a region which is displayed in B) showing an SEM cross-section of a piece of remaining Al_2O_3. C) SEM cross section at the intended nanogap region, when the Al_2O_3 etching step is integrated before the second pyrolysis, showing the merged first and second PyrC layer. Scalebars are 500 µm, 1 µm and 1 µm for A-C respectively.

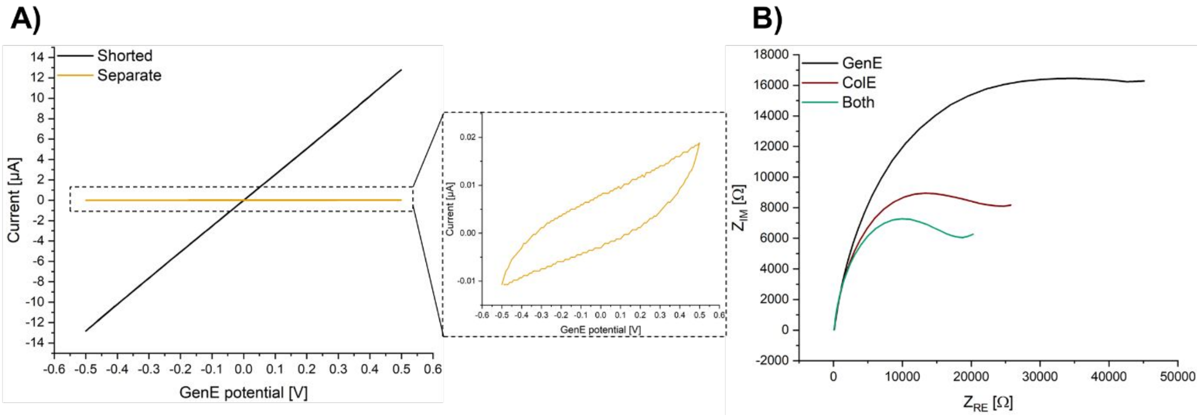

Figure S4: A) 2-electrode CVs with the working electrode terminal of the potentiostat connected to the bottom PyrC layer and the reference and counter electrode terminal connected to the top PyrC electrode. The measurement was performed in air from -0.5 V to 0.5 V at a scan rate of 100 mV s^{-1}. The shorted SLNE show clear resistive behavior while the electrodes with electrically separate terminals show a more capacitive response and much lower currents. B) EIS performed from 1 MHz to 0.1 Hz at open circuit potential with a 0.01 V AC perturbation using a buffer, with pH=7.4, consisting of 50 mM

K_2HPO_4/KH_2PO_4 (phosphate buffer) and 100 mM KCl (electrolyte) in DI water along with the electroactive species $[Fe(CN)_6]^{3-}/[Fe(CN)_6]^{4-}$ at an equimolar concentration of 0.5 mM. The working electrode terminal of the potentiostat was connected to the GenE, ColE and both GenE and ColE simultaneously. Connecting both GenE and ColE corresponds to connecting either electrode in parallel verifying separation.

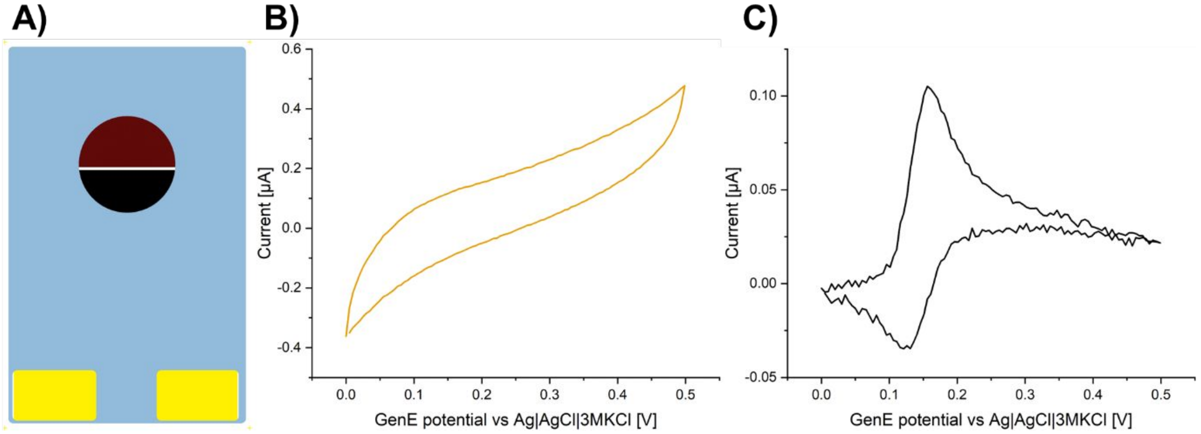

Figure S5: A) Top view schematic of design of macroelectrodes with one half of the region exposed to the same processing as the SLNE GenE and the other half to the same processing as ColE. The entire region is 12.56 mm^2 and the macroelectrode with the GenE layer PyrC (MacroGenE) is 4.94 mm^2. B) CV from 0 V to 0.5 V with a scan rate of 50 mV s^{-1} in nitrogen bubbled 1X PBS solution for the MacroGenE showing the capacitive background current. C) Background subtracted CV from 0 V to 0.5 V with a scan rate of 50 mV s^{-1} in nitrogen bubbled 1X PBS solution with the addition of 10 µM DA for the MacroGenE. The anodic peak current determined from this CV was used to calculate AF_{SEN}.

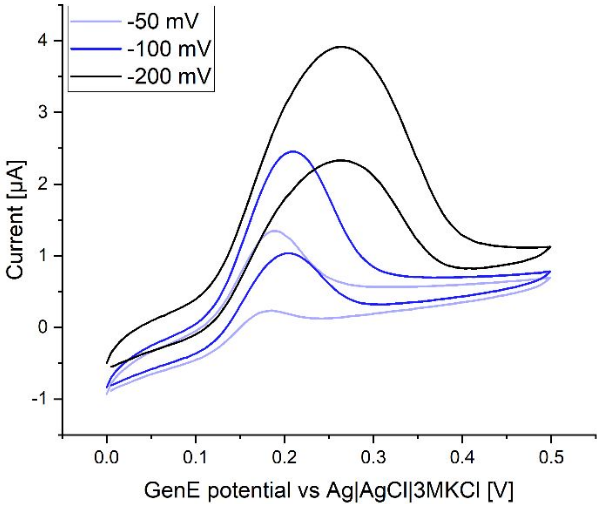

Figure S6: CV with SLNE-M for GenE current response in DRC mode with varying offset potentials. Measurements were performed in nitrogen bubbled 1X PBS with the addition of 50 µM DA using a Ag|AgCl|3MKCl RE and Pt wire CE and the 2nd scan is displayed.

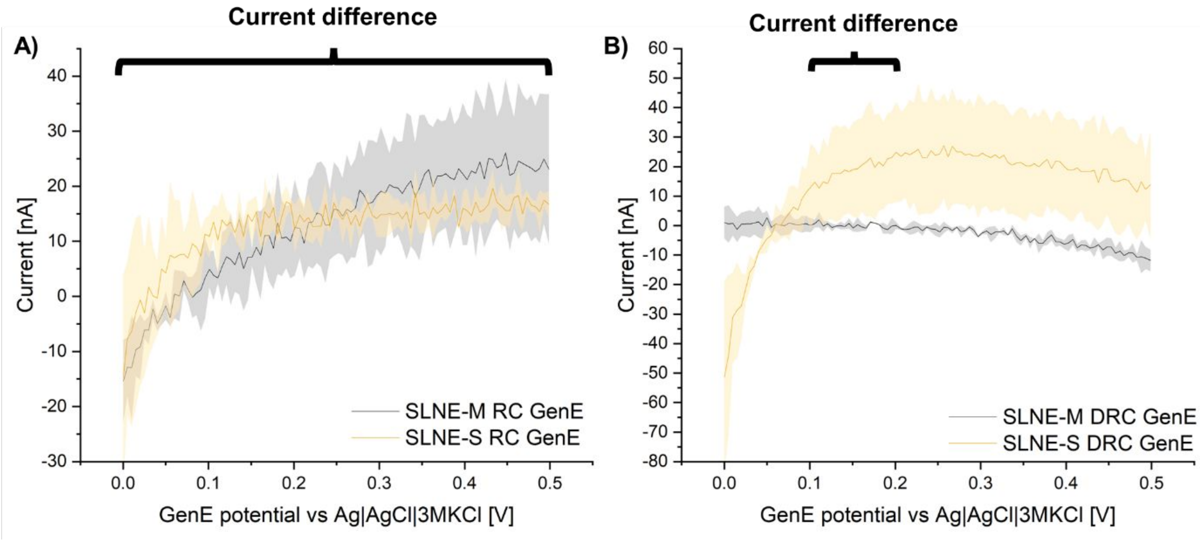

Figure S7: Background subtracted blank measurements using CV in either A) RC mode or B) DRC mode for SLNE-M and SLNE-S. Only the forward part of the scan is displayed. Measurements were performed in nitrogen bubbled 1X PBS using a Ag|AgCl|3MKCl RE and Pt wire CE and the 5th scan is displayed. The voltage range over which the current is measured is indicated at the top of the plots. The shaded region corresponds to the standard deviation between chips with n=3.

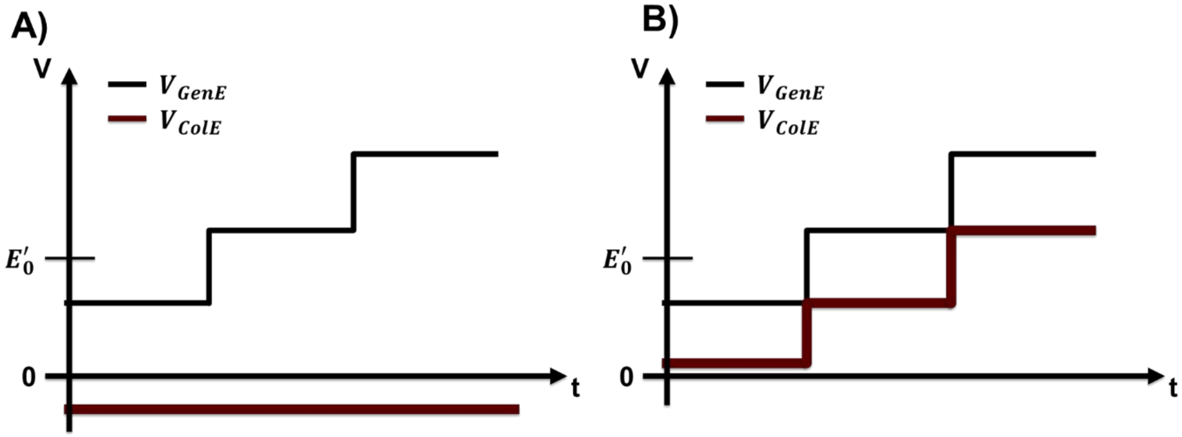

Figure S8: Simplified schematic of multistep CA corresponding to the steps 0.1 V, 0.2 V and 0.3 V for the measurements performed in Fig. 7 and 8 for A) RC mode and B) DRC mode. The formal potential of DA has been indicated on the y-axis.

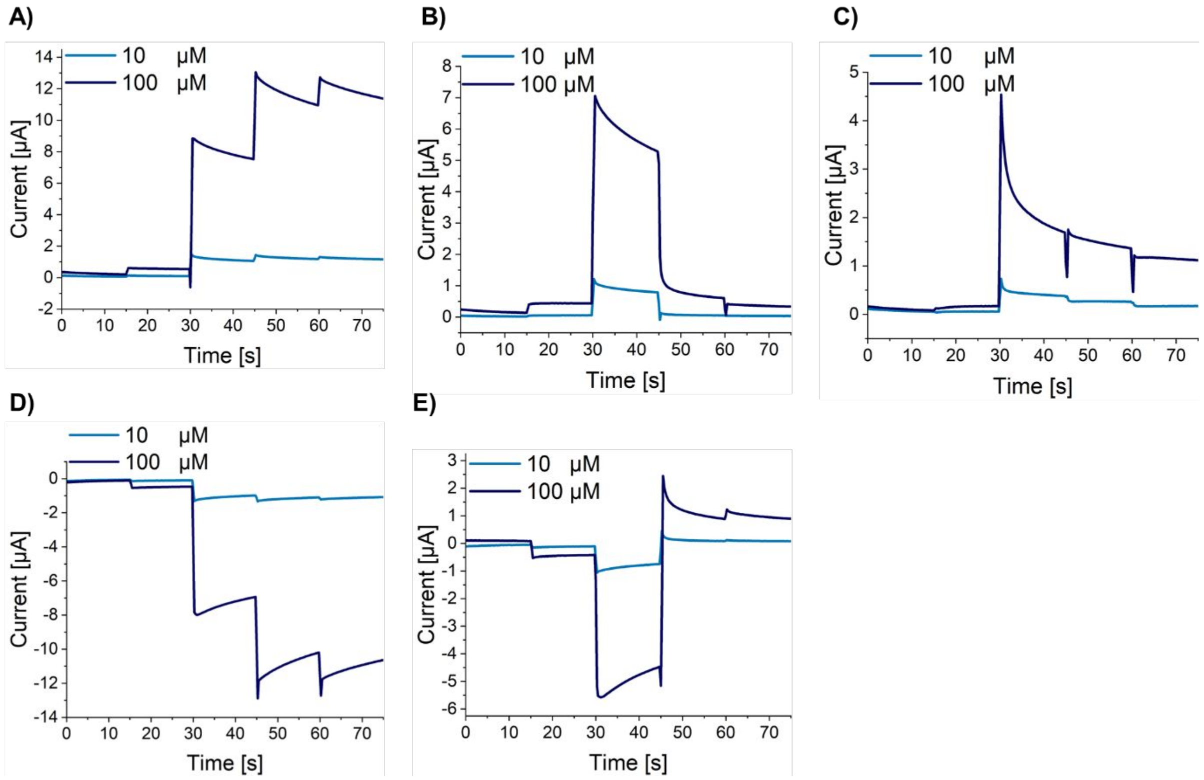

Figure S9: High concentration measurements corresponding to the experiments shown in Fig. 7 where A) is the RC mode GenE response, B) DRC mode GenE response, C) nonRC mode GenE response, D) RC mode ColE response and E) DRC mode ColE response.

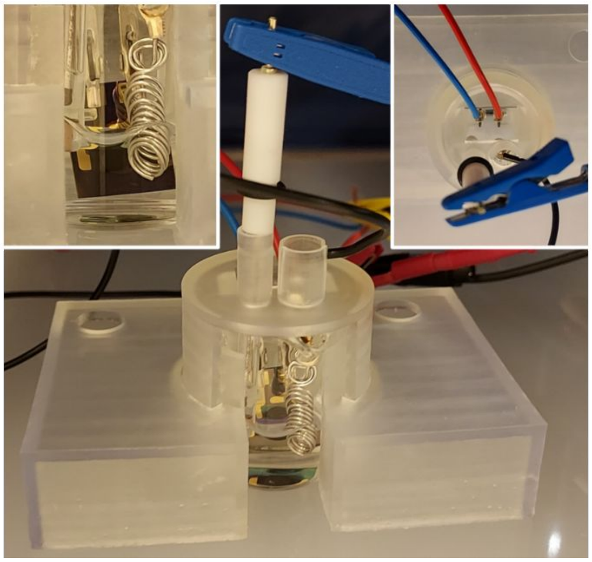

Figure S10: Electrochemical setup used during experiments with custom-made 3D printed electrochemical platform allowing chip insertion into glass beakers.
